# Crosstalk between YAP/TAZ and ERα in mechanical and hormonal signaling in the skeletal system: YAP/TAZ-ERα in the skeletal system

**DOI:** 10.3724/abbs.2025186

**Published:** 2025-10-27

**Authors:** Ruiying Han, Tianyi Wang, Yikai He, Ding Bai, Jing Xie, Yongwen Guo

**Affiliations:** 1 State Key Laboratory of Oral Diseases & National Center for Stomatology & National Clinical Research Center for Oral Diseases & Department of Orthodontics West China Hospital of Stomatology Sichuan University Chengdu 610041 China; 2 State Key Laboratory of Oral Diseases & National Center for Stomatology & National Clinical Research Center for Oral Diseases West China Hospital of Stomatology Sichuan University Chengdu 610041 China

**Keywords:** YAP/TAZ, ERα, mechanical signaling, estrogen, bone remodeling/bone biology

## Abstract

Bone remodeling represents a dynamic equilibrium orchestrated by mechanobiological and endocrine signals, with YAP/TAZ and ERα emerging as pivotal regulators of skeletal adaptation. YAP/TAZ functions as the central mechanotransduction hub of the Hippo pathway, converting biomechanical cues, including microenvironment matrix stiffness and shear stress, into osteogenic transcriptional programs. Concurrently, ERα integrates both mechanical stimuli and estradiol (E2) signaling to coordinate osteoblast-osteoclast coupling through the transcriptional regulation of RUNX2 activity and RANKL suppression. Although increasing evidence suggests that these two systems might engage in functional crosstalk, there is still no consensus on this issue. This review synthesizes the current understanding of YAP/TAZ-ERα interactions across three dimensions: (1) mechanohormonal integration in skeletal remodeling, (2) context-dependent reciprocity in breast carcinogenesis, and (3) tissue-specific regulatory paradigms in extra-skeletal systems. Key findings reveal that YAP/TAZ and ERα exhibit both synergistic cooperation (enhanced osteogenic differentiation via promoter co-occupancy) and pathway antagonism (competitive TEAD binding), with their interaction dynamics being critically shaped by the cellular microenvironmental context. Notably, mechanical potentiation of ERα transcriptional activity requires YAP/TAZ co-activation in bone mesenchymal stem cells, whereas estrogen signaling modulates YAP mechanosensitivity through cytoskeletal remodeling. These mechanistic insights indicate that the YAP/TAZ-ERα axis is a promising therapeutic target for osteoporotic bone loss, particularly in alveolar bone preservation. By bridging endocrine and mechanobiological perspectives, this work provides a conceptual framework for developing combinatorial therapies that simultaneously address hormonal imbalance and mechanical insufficiency in skeletal pathologies.

## Introduction

Bone remodeling represents a lifelong physiological process essential for skeletal maintenance and renewal [ 1, 2] . This dynamic equilibrium relies on the coordinated activities of osteoblasts and osteoclasts, which are meticulously regulated by biophysical signals, biochemical signals and their interactions. Mechanical signal transduction converges on the Hippo pathway effectors Yes-associated protein (YAP) and transcriptional co-activator with PDZ-binding motif (TAZ) molecular rheostats that translate biomechanical cues ( *e*. *g*., cell density, cell area, tissue stretch, shear forces, and matrix stiffness) into transcriptional programs that govern cell behaviors, including proliferation and differentiation [ 3, 4] . Physiological mechanical loading promotes bone anabolism through YAP/TAZ activation, whereas disuse-induced unloading precipitates rapid bone loss, underscoring their mechanosensitive regulation of skeletal integrity [5]. Among the biochemical signals, estrogen has emerged as a pivotal modulator of bone metabolism. Estrogens can be classified into a series of subtypes, of which estradiol (E2) has recently shown great potential as a major circulating estrogen in the vertebrate skeleton [6]. Research has indicated that endogenous E2 exerts dual regulatory effects through estrogen receptors (ERs), promoting osteoblastic bone formation via enhanced proliferation and survival of osteoprogenitor cells while suppressing osteoclastic bone resorption through inhibition of RANKL-mediated differentiation and induction of osteoclast apoptosis [ 6, 7] . Notably, emerging evidence highlights the critical interplay between hormonal regulation and mechanical signaling, an indispensable determinant of skeletal adaptation to environmental biophysical stimuli [8]. The ER family, comprising the nuclear receptors ERα/ERβ and the membrane-associated G protein-coupled estrogen receptor (GPER), demonstrates functional specialization in bone homeostasis [9]. ERα has been identified as a central integrator of both hormonal and mechanical signals, orchestrating bone remodeling through multiple mechanisms: (1) modulating RUNX2 transcriptional activity to direct osteoblast differentiation; (2) suppressing mesenchymal-derived osteoclastogenic factors; and (3) enhancing osteoclast apoptosis via nongenomic pathways [10].

The emerging paradigm of YAP/TAZ-ERα crosstalk presents a compelling mechanistic link between the endocrine and biomechanical regulation of bone remodeling. Current evidence suggests that (1) both systems converge on osteoblast/osteoclast lineage commitment; (2) ERα activation modulates YAP/TAZ nuclear translocation; and (3) mechanical stimuli may influence ER signaling efficacy. This review aims to systematically explore the interplay between ER signaling and YAP/TAZ mechanotransduction in bone metabolism, with a particular emphasis on their synergistic/antagonistic interactions in osteoporotic bone loss. A comprehensive literature search was conducted across the Medline, Embase, and Web of Science databases (from inception to May 2025) using the following Boolean query: [(E2 OR estrogen OR ERα OR “estrogen receptor α”) AND (YAP OR YAP/TAZ)]. By elucidating these molecular relationships, we aimed to identify novel therapeutic targets for maintaining alveolar bone mass in osteoporosis and provide potential therapeutic strategies for bone-related pathologies.

## YAP/TAZ as Mechanotransduction Orchestrators in Skeletal Adaptation

### Transduction mechanism by which YAP/TAZ respond to mechanical signals

YAP/TAZ transcriptional coactivators serve as central mechanotransporters that coordinate bone remodeling through the dynamic regulation of osteogenic programs. These evolutionarily conserved proteins interact with TEAD transcription factors to regulate cellular quiescence-proliferation decisions, stem cell maintenance, and tissue morphogenesis [ 11, 12] . In addition to developmental regulation, emerging evidence positions YAP/TAZ as multimodal integrators of mechanical and biochemical signals in bone homeostasis, modulating six fundamental processes: (1) osteoprogenitor proliferation/differentiation; (2) matrix mineralization dynamics; (3) cytoskeletal reorganization; (4) mechanoadaptive transcriptional programs; (5) anti-apoptotic signaling; and (6) osteocyte-osteoclast crosstalk [ 3, 13, 14] . The functions of YAP/TAZ are largely controlled by the Hippo signaling pathway, which consists of multiple Ste20 family kinases (MST1/2, TAO), which phosphorylate and activate the LATS1 and LATS2 serine/threonine-protein kinases. Multiple accessory proteins, such as protein salvador homologue 1 (SAV1), MOB kinase activator 1A (MOB1), and merlin (NF2), promote LATS1/2 activation by upstream kinases. Once activated, LATS1/2 phosphorylates YAP/TAZ at multiple sites to promote its nuclear export and/or degradation through the ubiquitin proteasome system [ 15, 16] . Mechanical signals modulate YAP/TAZ activity through the actin cytoskeleton and the Hippo pathway. Specifically, the functions of YAP/TAZ are directly influenced by F-actin-mediated tension generated by mechanosensors, including integrins, E-cadherin, and mechanosensitive ion channels. Under mechanical stress, these sensors not only reinforce the cellular structure by stabilizing associated actin networks but also modulate YAP/TAZ signaling. This section summarizes how mechanical cues, via various mechanosensors, facilitate F-actin accumulation and promote YAP/TAZ nuclear translocation.

**Figure 1:**
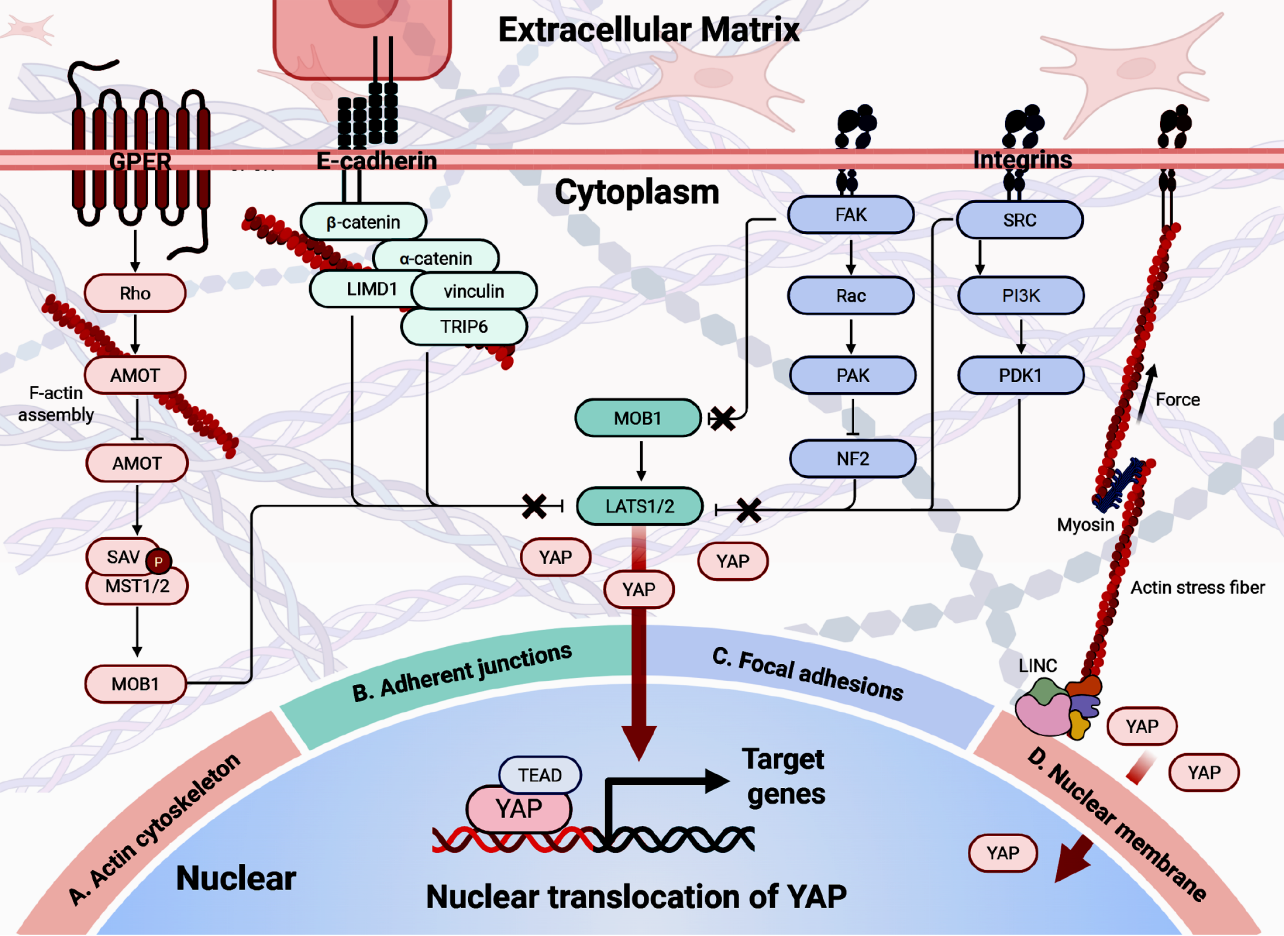
Figure 1 YAP mediates cellular mechanical signals (A) Actin cytoskeleton. External forces are delivered via the extracellular matrix to integrins and adhesion proteins and induce F-actin assembly. F-actin prevents AMOT from binding to SAV-MST1/2. This prevents SAV-MST1/2 from activating LATS1/2 via MOB1, and YAP/TAZ cannot be phosphorylated by LATS, thus allowing them to freely enter the nucleus. (B) Adherent junctions. E-cadherin binds β-catenin to α-catenin and actin stress fibers under high mechanical tension/low cell density stimulation. The conformational change of β-catenin increases its binding to LIMD1 and vinculin. LIMD1 inhibits LATS1/2, and vinculin recruits TRIP6 to compete with MOB1 to bind to LATS1/2. The inhibition of LIMD1 and TRIP6 on LATS1/2 leads to the free translocation of YAP/TAZ to the nucleus. (C) Focal adhesions. When integrins bind to a harder extracellular matrix, they trigger the activation of focal adhesion signals driven by focal adhesion kinase (FAK) and SRC tyrosine kinase. FAK and SRC can directly phosphorylate MOB1 and LATS1/2, respectively, to inhibit their activity. They also enhance the nuclear translocation of YAP by inhibiting the activity of LATS1/2 via other signaling pathways, such as the FAK-SRC-PI3K-PDK1 pathway. (D) Nuclear membrane. Increased matrix stiffness causes the focal junctions of the cell membrane to connect to the LINC complex on the nuclear membrane via F-actin, leading to an increase in contraction force and flattening of the nucleus. Stronger permeability of nuclear pores leads to an increase in nuclear YAP level.

YAP/TAZ-mediated osteogenic differentiation occurs in response to diverse biophysical and biochemical stimuli. Among these factors, matrix stiffness and surface topography have been the most extensively investigated. A stiff matrix that promotes cell spreading and adhesion enhances focal adhesion (FA) formation and cytoskeletal stress fiber assembly, facilitating YAP/TAZ nuclear translocation through both Hippo-dependent and Hippo-independent mechanisms. This nuclear translocation and accumulation promote osteogenic differentiation while suppressing adipogenesis, which is achieved through coordinated activation of the transcription factor RUNX2 and inhibition of downstream signaling, such as peroxisome proliferator-activated receptor gamma (PPARγ) [ 17, 18] . Conversely, a soft matrix that impedes cell spreading and adhesion reduces FA formation and cytoskeletal tension, leading to decreased YAP/TAZ activity and consequent inhibition of osteogenic differentiation with concomitant promotion of adipogenic differentiation. This section delineates the mechanoregulatory architecture of YAP/TAZ through four principal mechanosensory modules ( Figure 1).

#### Actin cytoskeleton

Cells sense mechanical signals through extracellular and intracellular receptors, including those associated with adhesion junctions, FA, and nuclear membranes. Cellular mechanical signals initiate actin networks that transduce extracellular forces into intracellular biochemical signals [ 3, 16] . In general, increasing F-actin levels promote YAP/TAZ nuclear localization, and loss of F-actin causes YAP/TAZ to accumulate in the cytoplasm. The actin polymerization status serves as a rheostat for YAP/TAZ activity, which increases stress fiber formation and promotes nuclear accumulation through angiomastin-like protein (AMOT) sequestration [ 19, 20] . When actin bundles depolymerize, AMOT is free to bind to SAV-MST1/2 and LATS1/2, and SAV-MST1/2 indirectly activates LATS1/2 via MOB1, thus inhibiting the nuclear localization of YAP/TAZ. When AMOT binds to actin polymers, YAP/TAZ cannot be inhibited by AMOT and can freely enter the nucleus [ 19, 20] .

#### Adherent junctions

Cell-cell contact inhibition is the first factor that has been shown to regulate the Hippo pathway. In epithelial tissues, tight junctions exist between cells, and the tension generated by the contractile forces among individual cells is transmitted between cells through cell-cell junctions called adherent junctions. Adherent junctions are composed of transmembrane cadherin-catenin complexes. The cytoplasmic region of cadherins binds to β-catenin, which interacts with F-actin to connect itself to the actin cytoskeletal network. Intercellular mechanical coupling regulates Hippo signaling via tension-sensitive scaffolds through E-cadherin complexes, which bind β-catenin to α-catenin and actin stress fibers under high mechanical tension/low cell density stimulation [21]. Under high mechanical strain, β-catenin conformational changes promote LIM domain-containing protein 1 (LIMD1)-mediated LATS inhibition and TRIP6-MOB1 competition, enabling YAP nuclear translocation [21]. LIMD1 inhibits LATS1/2, and vinculin recruits TNF receptor binding factor 6 (TRIP6) to compete with MOB1 for binding to LATS1/2. The inhibition of LIMD1 and TRIP6 by LATS1/2 leads to the free translocation of YAP/TAZ to the nucleus [ 22, 23] . Conversely, static loading conditions favor LATS activation through MOB1 binding, maintaining YAP cytoplasmic retention [3].

#### Focal adhesions

Integrins, a typical type of FA, are transmembrane proteins connecting cells to the matrix that interact with the microenvironmental matrix via the extracellular domain while interacting with proteins via the intracellular domain. Stiffer substrates result in stronger binding between focal adhesions and stress fibers and increased activation of the FAK kinase YAP/TAZ. Integrin-mediated mechanotransduction converges on YAP regulation through dual kinase cascades. When integrins bind to the stiffened extracellular matrix, stiffness-dependent FAK/SRC activation is triggered [24]. FAK/SRC directly phosphorylates YAP at tyrosine residues to increase transcriptional activity [24]. They also enhance the nuclear translocation of YAP by inhibiting the activity of LATS1/2 and MOB1 via direct phosphorylation or other signaling pathways, such as the PI3K-PDK1 signaling [ 3, 25] .

#### Nuclear membrane

Emerging evidence identifies nuclear mechanosensing as a distinct regulatory layer. This mechanostructural regulation operates independently of Hippo kinases. A high mechanical stress microenvironment, including a highly stiffened extracellular matrix and tensile stress, activates the nuclear translocation of YAP/TAZ and promotes stem cell differentiation [3]. Matrix stiffness governs nuclear morphology through the linker of the nucleoskeleton and cytoskeleton (LINC). F-actins act to connect focal adhesions to the LINC complex on the nucleus, thus inducing nuclear flattening and YAP influx via nuclear pores, whereas soft substrates lead to cytoplasmic retention of YAP via nuclear pore compression [ 3, 26] .

### YAP/TAZ regulates bone development and homeostasis by affecting cell fates in bone tissue

#### YAP/TAZ regulates the differentiation and proliferation of osteoblasts

YAP/TAZ influences mesenchymal stem cell (MSC) lineage commitment, promoting osteoblastogenesis over adipogenesis [ 27, 28] . However, Sox2-controlled YAP independently favors adipogenic over osteogenic differentiation in MSCs [29]. Interestingly, Vestigial-like family member 4 (VGLL4) directly counteracts TEAD-mediated inhibition of Runx2, promoting osteoblast-lineage differentiation. This VGLL4/TEAD osteogenic function operates independently of YAP and the Hippo signaling pathway [30].

YAP/TAZ exert stage-specific effects on osteoblast lineage cells [31]. Mouse models reveal distinct roles depending on the osteoblast differentiation stage. Double knockout (DKO) of YAP/TAZ in Osx ^+^ or Prx1 ^+^ osteoprogenitors causes embryonic lethality due to ribcage malformation or hemorrhage, respectively. Single deletion of YAP or TAZ in Osx ^+^ cells or DKO in mature osteoblasts/osteocytes decreases bone mass and reduces bone mass, which is correlated with increased osteoclast activity and decreased osteoblastogenesis [32]. Mechanistically, YAP/TAZ suppress canonical Wnt signaling and Runx2 activity in osteoblast progenitors. Consequently, their deletion enhances osteoblast differentiation *in vitro*. Consistent with these findings, the deletion of YAP and TAZ from osteoprogenitor cells increased osteoblast differentiation *in vitro* [ 31, 33] . Conversely, deletion of YAP and TAZ by Dmp1-Cre in mature osteoblasts and osteocytes reduced osteoblast numbers and bone formation while increasing osteoclast numbers without altering key regulators such as RANKL, osteoprotegerin (OPG), or sclerostin (SOST). These findings indicate that YAP/TAZ inhibit differentiation towards the osteoblast lineage in progenitors but promote bone formation and suppress resorption in mature osteoblasts/osteocytes.

In progenitors, YAP/TAZ inhibition of Wnt signaling and Runx2 significantly impairs osteogenic potential. In mature osteoblasts, however, they enhance bone-forming activity. This stage-specific regulation involves multiple mediators: integrin αv responds to mechanical stimulation by activating YAP/TAZ via the Src-JNK-YAP/TAZ pathway [34]. Osteogenic differentiation enhances the regulatory function of CTHRC1 on TAZ in periodontal ligament stem cells, suggesting implications for periodontal regeneration [35]. YAP/TAZ also promotes osteoblast differentiation by inducing LPR6 and DVL3 expression, indirectly modulating Wnt/β-catenin signaling [36]. BMP-2 signaling-associated Smad1/5/8 complexes synergize with YAP/TAZ to activate osteogenic genes, while the flavonoid fisetin suppresses this interaction and osteogenic expression [37]. Additionally, the AMPK axis promotes osteoblast lineage differentiation independently of the Hippo cascade and YAP/TAZ [38].

#### YAP/TAZ regulates the differentiation and proliferation of osteoclasts

The osteoblast-osteoclast balance is crucial for bone homeostasis, with emerging evidence identifying YAP as a key osteoclastogenesis regulator. *YAP* knockdown in bone marrow-derived macrophages (BMMs) inhibits multinucleated osteoclast formation [39]. Similarly, verteporfin (VP), which disrupts YAP-TEAD binding, reduces osteoclastogenesis. YAP activation also upregulates GDF15, promoting osteoclast differentiation in RAW264.7 cells [40]. MST2 deletion enhances osteoclastogenesis via the RANKL pathway, leading to bone loss [41]. Osteoclast-specific *TAZ* knockout studies revealed that TAZ interacts with transforming growth factor β-activated kinase-1 (TAK1) to inhibit NF-κB signaling and osteoclastogenesis [42]. Furthermore, downregulation of TEAD1 decreases OPG activity and stimulates osteoclast lineage differentiation [43].

#### YAP/TAZ and osteocytes

YAP/TAZ plays a central role in perilacunar/canalicular network formation and osteocyte-mediated bone remodeling [32]. As osteocytes are mechanosensitive and YAP/TAZ act as the central mechanotransducer, they likely mediate osteocyte-driven bone remodeling. TGF-β induces matrix-remodeling enzyme expression in osteocytes, while suppressing YAP/TAZ activation in mouse osteocytes (using 8-kb DMP1-Cre) inhibits this expression [32]. In MLO-Y4 osteocyte-like cells, shear stress-activated YAP/TAZ participates in the mechanoinduction of the chemokines macrophage colony-stimulating factor 1 (MCSF) and C-X-C motif chemokine 3 (CXCL3) [44]. Collectively, this evidence demonstrates that YAP/TAZ mediate the ability of osteocytes to promote osteoblast activity and suppress osteoclastogenesis.

In conclusion, YAP/TAZ serve as critical mechanosensors and transcriptional regulators in bone remodeling, orchestrating osteoblast differentiation and osteoclastogenesis in response to mechanical and biochemical cues. Their activity is precisely regulated by substrate stiffness, cytoskeletal tension, and crosstalk with key pathways, including the Wnt/β-catenin, BMP, and RANKL signaling pathways. Further research is needed to elucidate their spatiotemporal regulation and therapeutic potential in bone-related diseases.

### YAP/TAZ interact with other pathways in bone development and homeostasis

YAP/TAZ occupies a central position within the complex cellular signaling network and exhibits significant crosstalk with multiple pathways. Evidence indicates that diverse signaling pathways converge to regulate YAP/TAZ-induced osteogenesis.

#### The interplay of YAP/TAZ and Wnt signaling

The interaction between YAP/TAZ and Wnt signaling is bidirectional, with significant implications for normal bone growth, gene expression and malignancies. β-Catenin, an essential mediator of canonical Wnt signaling, is transcriptionally linked to bone formation [45]. Canonically, Wnt signaling activation inactivates the cytoplasmic β-catenin degradation complex containing GSK-3β. This leads to β-catenin accumulation, nuclear translocation and binding to transcriptional partners to exert its effects. In diverse cell types, YAP/TAZ serves as a component of the β-catenin destruction complex in the absence of Wnt stimulation. Upon Wnt activation, YAP/TAZ is released from the complex, translocates into the nucleus, and contributes to β-catenin stabilization [46]. Specifically, YAP binds the cytoplasmic destruction complex with β-catenin; Wnt signal activation triggers its dissociation, enabling the nuclear translocation and transcriptional activation of both YAP and β-catenin [47]. Alternatively, Wnt ligands can activate YAP independently of β-catenin [48]. Furthermore, an interaction may occur between the YAP-TEAD and β-catenin-TCF complexes, forming a transcriptional unit [49].

The Wnt pathway modulates YAP/TAZ activity through diverse, often opposing, mechanisms, reflecting context-dependent responses. This crosstalk critically influences MSC lineage commitment. For instance, exosomal Wnt5A regulates YAP to promote a tumor-supportive MSC lineage, while Wnt1 enhances bone formation via YAP and BMP in osteoblasts [50]. The Wnt3A/YAP/TEAD and Wnt3A/PP1A/TAZ axes promote MSC osteoblast-lineage differentiation [51]. Additionally, noncanonical Wnt ligands like Wnt4 promote YAP/TAZ activation via an alternative axis (Wnt-FZD/ROR-Gα12/13-Rho-Lats1/2), independent of β-catenin or LRP5/6 coreceptors during osteogenesis [46].

In summary, the regulation between YAP/TAZ and Wnt signaling is bidirectional, and their interplay has significant implications for bone tissue physiology and pathology. This interplay offers novel therapeutic targets for related diseases.

#### The interplay of YAP/TAZ and TGF-β signaling

TGF-β is a multifunctional cytokine regulating numerous developmental and homeostatic processes. TGF-β initiates cellular responses by binding to cell surface receptor complexes, triggering activation of intracellular Smad signaling molecules [52]. TGF-β2 enhances the phosphorylation of Smad3 and facilitates the nuclear accumulation of p-Smad3, and then enhances the cytoskeleton and focal adhesion plaque at transcriptional level [53]. YAP/TAZ binds to Smad transcription factors, the canonical mediators of profibrotic TGF-β responses. TGF-β-induced canonical Smad2/3 signaling is mechanoregulated by extracellular matrix (ECM) stiffness via control of Smad2/3 localization, a process requiring YAP and TAZ [54]. Furthermore, the BMP-2/Smad1/5/8/YAP/TAZ axis specifies osteoblast lineage commitment in myoblasts [37]. YAP/TAZ also modulates BMP and TGF-β transcriptional responses through interactions with SMAD1/5 [55]. In neural crest stem cells (NCSCs), LATS1/2-regulated YAP modulates the TGF-β-driven epithelial-to-mesenchymal transition [56]. Additionally, reduced YAP/TAZ levels in human dermal fibroblasts activate activator protein 1 (AP-1), inducing Smad7 expression and subsequent suppression of the TGF-β pathway [57]. Collectively, these findings highlight the complex and dynamic reciprocal regulation between YAP/TAZ and TGF-β signaling in governing mesenchymal and skeletal lineage differentiation and function.

## Estrogen Signaling as the Key Hormonal Orchestrators in Skeletal Adaptation

### E2 and ERs signaling transduction mechanism

Estrogens represent a group of estrogen hormones, including estrone, estriol and estradiol, among which estradiol secreted by the granulosa cells and corpus luteum of the ovarian follicles is the major circulating estrogen in humans [9]. Typical ERs include nuclear receptors ERα and ERβ, as well as G protein-coupled ER (GPER) located in cell membrane [9].

#### Direct and indirect genomic signaling

Nuclear receptors ERα and ERβ mediate direct and indirect genomic signaling of estrogen. In direct genomic signaling, ligand-activated ER is isolated from its partner heat shock protein and binds directly to the estrogen response element (ERE) in the promoter of target genes, then interacting with cofactors (coactivators or corepressors) to regulate gene expression [ 58, 59] . For the indirect genomic signaling, ligand-activated ERs do not bind to DNA directly, but rather through protein-protein interactions with other classes of transcription factors at their respective response elements. For example, estrogen-ER complex interacts with FBJ murine osteosarcoma viral oncogene homologue (FOS) and Jun proto-oncogene (JUN) proteins at the AP-1 binding sites in genes encoding ovalbumin, insulin-like growth factor 1 (IGF1), collagenase, cyclin D1 (CCND1) and choline acetyltransferase. The result depends on the ER subtype and type of the ligand [60].

#### Indirect non-genomic signaling

Membrane receptor GPERs mediate indirect non-genomic signaling through second messenger systems ( *e*. *g*., cAMP) and kinase activation cascades, enabling rapid cellular responses independent of nuclear translocation. This mechanism typically involves activation of signal transduction, such as the generation of intracellular second messengers and activation of protein kinases in the signaling cascade, leading to indirect changes in gene expression [ 61, 62] .

#### Ligand-independent signaling

**Figure 2:**
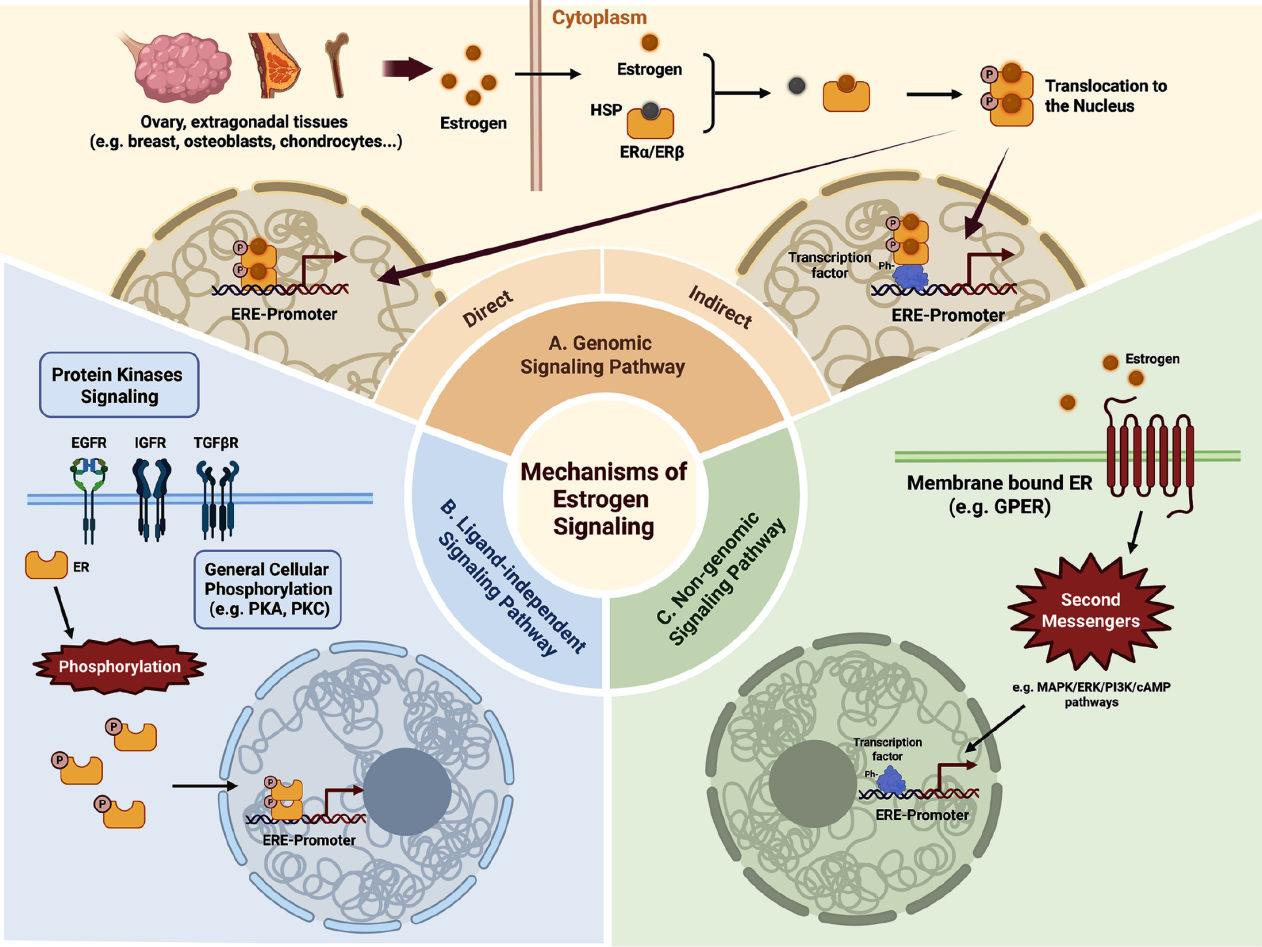
Figure 2 The signal transduction mechanism of E2 and ERs in the skeletal system (A) The nuclear receptors ERα and ERβ mediate the direct and indirect genomic signaling of estrogen. (B) The ligand-independent signaling pathway is triggered primarily by the phosphorylation of specific residues in ERs. (C) Membrane receptor GPERs mediate indirect non-genomic signaling of estrogen.

Notably, ERs also exhibit ligand-independent signaling pathway via phosphorylation of specific serine/tyrosine residues [63]. This ligand-independent ER activation is primarily triggered by the phosphorylation of specific residues itself like serine and tyrosine, and thus requires the involvement of phosphorylation-regulating molecules, including kinases (PKA, PKC, and MAPK), growth factors (IGF-1, EGF, and TGF-β), inflammatory cytokines (IL-2), and mechanical stress-responsive pathways [64]. A summary of E2 and ERs signal transduction mechanism is shown in Figure 2.

### ERα as a central mediator of mechanical and estrogenic regulation in skeletal homeostasis

Mechanical stimulation and E2 are two key factors in the regulation of bone remodeling, both converging on ERα to coordinate bone growth, maturation, and homeostasis [10]. Among ERs, as the dominant ER subtype in bone, ERα plays an indispensable role in osteogenic responses to mechanical stress, as multiple experiments have shown that no proliferation reaction occurred in the osteoblastic precursor cells when ERα were blocked or knocked out [65]. Notably, ERα can drive osteogenic responses to mechanical stimuli even in a ligand-independent manner [63]. Mechanical loading triggers ERα phosphorylation via extracellular signal-regulated kinase (ERK) in osteocytes, initiating downstream osteogenic programs [66]. This function of ERα does not require the existence of estrogen and may be mediated by the activation function-1 (AF-1) domain to stimulate gene transcription [67]. Genetic or pharmacological inhibition of ERα in bone mesenchymal stem cells (BMSCs) impairs mechanoadaptation, suppressing proliferation, alkaline phosphatase (ALP) activity, ERα expression, and F-actin cytoskeletal reorganization [68]. ERα orchestrates these processes through genomic transcriptional regulation and crosstalk with mechanosensitive pathways, including insulin-like growth factor (IGF) and Wnt/β-catenin signaling. ERα serves as a critical node for Wnt/β-catenin pathway activation—loss of ERα disrupts β-catenin nuclear translocation and subsequent transcription of osteogenic targets ( *e*. *g*., *Axin2*, and *Cyclin D1*) [69]. Pang *et al*. [70] demonstrated that the induction of osteogenic differentiation of BMSCs by quercetin (an estrogen analogue) could be inhibited by ERα inhibitors, underscoring ERα’s indispensable role in E2-mediated osteoblastogenesis. In the osteoclast lineage, ERα activation by E2 exerts bone-protective effects by antagonizing RANKL signaling. Dou *et al*. [71] revealed that E2-bound ERα inhibits NF-κB nuclear translocation in bone marrow macrophages (BMMs), thereby attenuating osteoclast differentiation. Collectively, ERα integrates mechanical and hormonal cues to promote osteoblast activity while suppressing osteoclastogenesis, ensuring net bone formation.

Various cell types including osteoblast progenitor cells, osteoclasts, and B lymphocyte are all involved in estrogen signaling for bone protection [72]. ERα differentially regulates trabecular and cortical bone through cell-type-specific mechanisms. In trabecular bone, ERα directly suppresses osteoclast resorption and modulates B lymphocyte-derived RANKL, a key contributor to postmenopausal trabecular bone loss [73]. RANKL expression in lymphocyte B contributes to the loss of trabecular bone caused by estrogen deficiency [ 74, 75] . In cortical bone, ERα-mediated effects on osteoblast progenitors indirectly attenuate resorption of the inner surface of the cortex through a mechanism of nonnuclear priming, and stimulate Wnt signaling and periosteal bone accumulation [ 76– 78] in response to mechanical stress. These spatially distinct effects highlight ERα’s ability to interpret microenvironmental cues ( *e*. *g*., cytokines, growth factors, mechanical strain) to tailor bone remodeling responses [72].

## Crosstalk between YAP/TAZ and ERα Signaling

A systematic search of the Medline, Embase, and Web of Science databases was performed for publications for studies up to May 2025 with no start date limited. Keywords used in databases searches were [(E2) OR (estrogen) OR (ERα) OR (estrogen receptor α)] AND [(YAP) OR (YAP/TAZ)]. A total of 285 relevant studies were retrieved after removing duplicate literature. The potential crosstalk between YAP/TAZ and ERα signaling needs to be urgently established due to the important cues revealed in the recent studies that emphasize the synchronous changes between them. To interpret the potential interaction between Hippo-YAP and E2-ERα signaling, we have divided this intimate linkage into three parts for detailed description as follows.

### Mechanohormonal integration between YAP/TAZ and ERα signaling in skeletal homeostasis

Emerging evidence suggests YAP/TAZ and ERα converge as critical regulators of bone mechanoadaptation, although their molecular crosstalk remains underexplored in skeletal biology. This section delineates the current understanding of their potential interactions within the bone remodeling paradigm, where mechanical and endocrine signals coordinate to maintain skeletal integrity.

**Figure 3:**
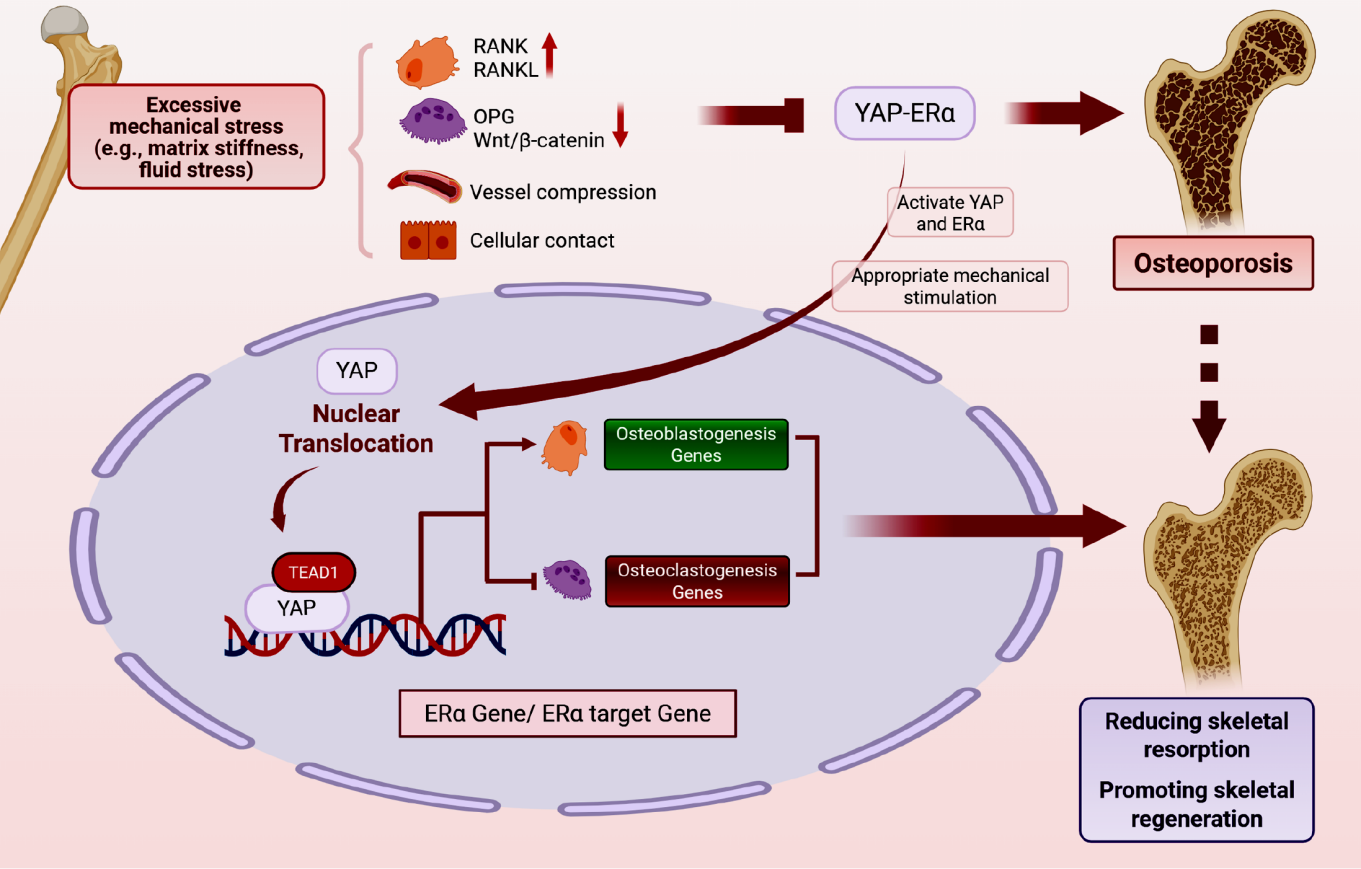
Figure 3 Reciprocal regulation between Hippo-YAP and ERα signaling in the skeletal system Mechanical and estrogen signals coordinate to maintain skeletal homeostasis. When mechanical stimulation is absent or below a certain threshold, it leads to a significant decrease in bone mass and bone quality. When appropriate mechanical stimulation and estrogen signals exist, YAP/TEAD1 directly induces ERα to promote osteogenesis and inhibit the osteoclastic differentiation of BMSCs by directly binding TEAD1 to the ERα promoter.

The bone-protective effects of estrogen primarily manifest through ERα-mediated dual regulation: (1) enhancement of osteoblast lineage proliferation/survival via Wnt/β-catenin and MAPK pathways and (2) suppression of osteoclastogenesis through RANKL inhibition and pro-apoptotic signaling [10]. Mechanical stimulation is a key factor affecting bone remodeling. Proper mechanical stimulation promotes bone formation and inhibits bone resorption. When mechanical stimulation is absent or below a certain threshold, it leads to a significant decrease in bone mass and bone quality [5]. It has been proved that YAP/TEAD1 synergistically induces ERα to promote osteogenic differentiation of BMSCs via direct binding to the ERα promoter [51]. Crucially, ERα demonstrates mechanoresponsive properties, modulating Runx2 activity to orchestrate osteoblast differentiation under combined estrogenic and mechanical stimuli [79]. This integrated regulatory network coordinates with the role of Runx2 to prime osteoprogenitors for mitotic responses through GPR30 activation and Rgs2 suppression [80]. Parallel mechanotransduction mechanisms involve YAP/TAZ nuclear shuttling mediated by cytoskeletal tension and LATS kinase inhibition. Biomechanical loading induces characteristic YAP/TAZ nuclear accumulation, driving transcriptional programs for osteogenic differentiation and matrix mineralization. Intriguingly, experimental models reveal synergistic effects between estrogen and mechanical tension in enhancing both ERα expression and YAP-mediated osteogenesis [81]. The reciprocal regulation between Hippo-YAP and ERα signaling in skeletal system is illustrated in Figure 3.

Current limitations in bone-specific research necessitate extrapolation from other systems. The hepatic model demonstrates YAP/TAZ-ERα cooperativity in oxidative stress mitigation [82], while uterine studies reveal mechanical potentiation of YAP/TAZ-ERα pro-fibrotic signaling [83]. These tissue-specific paradigms highlight the contextual nature of YAP/TAZ-ERα interactions, underscoring the need for dedicated skeletal investigations to elucidate bone-specific regulatory logic.

### Reciprocal regulation between YAP/TAZ and ERα signaling in breast carcinogenesis

**Figure 4:**
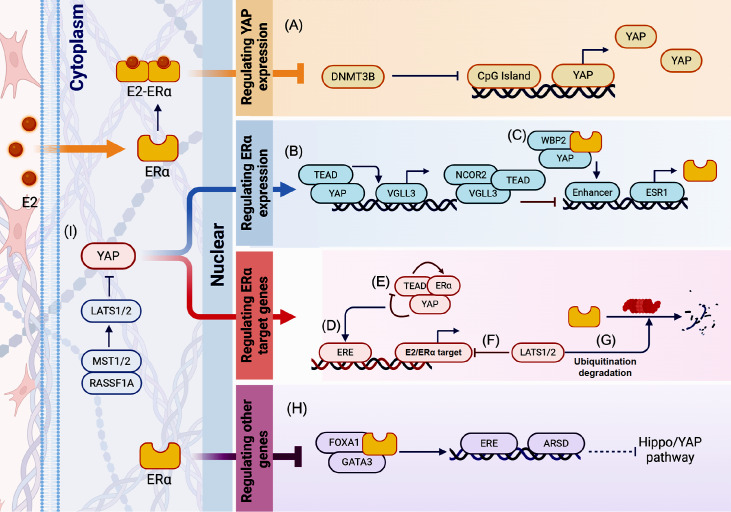
Figure 4 Reciprocal regulation between Hippo-YAP and ERα signaling in breast carcinogenesis The black arrow indicates synergy, and the red arrow indicates antagonism. Regulating YAP expression: (A) ERα combined with E2 can inhibit the activity of DNMT3B and reduce the degree of methylation of the YAP promoter, thus promoting YAP expression. Regulating ERα expression: (B) VGLL3, the target gene expression product of YAP, can compete with YAP/TAZ for binding to TEAD transcription factors and recruit the NCOR2 repressor to the superenhancer of the ESR1 gene, resulting in transcriptional silencing of ERα; (C) When the ER is activated by E2, WBP2 binds to YAP, which contains the WW domain, stimulating transcriptional activation of ERα. Regulating ERα target genes: (D) E2 promotes the binding of YAP1/TEAD4 to the enhancer of the ERα target gene, regulating enhancer activation and transcription of the ERα target gene; (E) TEAD physically interacts with ERα, increasing its occupancy of the target gene promoter/enhancer, whereas YAP inhibits ERα/TEAD interactions, reducing ERα occupancy at its target promoter/enhancer; and promoting the degradation of ERα by the proteasome; (F) LATS2 can co-localize with ERα within the nucleus, and LATS2 silencing increases the expression of ERα regulatory genes; (G) ERα is targeted for ubiquitination and degradation by DCAF1-dependent proteasomes in the presence of LATS. Regulating other genes: (H) ERα forms a complex with FOXA1 and GATA3, which activates the expression of ARSD gene and then activates YAP pathway. Left part: (I) RASSF1A inhibits the expression of ERα by promoting LATS to inhibit YAP, thus inhibiting ERα-dependent breast carcinogenesis.

Studies investigating the crosstalk between YAP/TAZ and ERα signaling in skeletal tissues remain scarce, while emerging evidence predominantly focuses on the crosstalk between ERα and YAP/TAZ in breast cancer tissues. ERα is expressed in 70% of breast cancers and is activated by binding to estrogen ligands such as E2, regulating various genes that control tumor cell survival and proliferation [ 84, 85] . Concurrently, the evolutionarily conserved Hippo pathway governs tissue homeostasis by modulating cell contact inhibition, organ size, and oncogenic transformation [86]. While YAP hyperactivation is protumorigenic in multiple malignancies ( *e*. *g*., hepatocellular, colorectal, and cervical cancers), its role in breast cancer exhibits context-dependent duality [ 87– 89] . Current findings reveal that ERα and YAP/TAZ engage in bidirectional regulation through direct transcriptional interplay, shared upstream modulators ( *e*. *g*., LATS1/2, MST1/2, and RASSF1A), and intermediate effectors like ARSD. Thus, ERα and YAP/TAZ exert an effect on each other through regulating YAP or ERα expression, ERα target gene expression, other genes' expression, and YAP nuclear translocation. However, emerging controversies underscore the complexity of this interaction. Figure 4 summarizes the reciprocal regulation between Hippo-YAP and ERα signaling in breast carcinogenesis.

#### Regulating YAP or ERα expression

ERα and YAP reciprocally modulate each other’s transcriptional activity. Muhammad *et al*. [90] demonstrated that by binding to E2, ERα suppresses the activity of DNA methyltransferase (DNMT3B) and reduces the methylation of the *YAP* promoter region, epigenetically upregulating YAP expression. Both YAP and ERα contain the WW domain. The WW domain enables the competitive binding to WBP2, a coactivator of ERα which triggers downstream signal transduction after nuclear translocation. Once ERα is activated by E2, WBP2 will bind to YAP and stimulate transcriptional activation of ERα, which is involved in breast cancer progression [91]. However, Ma *et al*. [92] suggested that YAP suppresses ERα expression in breast cancer. That is, the residual-like protein 3 (VGLL3), a target gene expression product of YAP, competes with YAP/TAZ for binding to TEAD transcription factors and recruits the NCOR2/SMRT repressor to the super-enhancer of the *ESR1* gene, resulting in transcriptional silencing of *ERα*.

#### Regulating ERα target genes

By binding to the estrogen response element (ERE) in the promoter of ERα target genes, cofactors exert effects on ERα target genes' expression. For example, Zhu *et al*. [93] identified that YAP1-TEAD4 complexes co-occupy ERα target enhancers, with E2 potentiating their association to activate enhancer-driven transcription and breast cancer progression. However, Li *et al*. [87] proved that YAP activation disrupts ERα transcriptional programs by dissociating ERα-TEAD complexes, promoting ERα proteasomal degradation. Another study suggests that LATS1/2 is required for the maintenance of ERα expression, and the loss of LATS1/2 disrupts *ESR1* mRNA and protein in breast, endometrial, and tubal epithelial cells in a manner dependent on LATS kinase activity and YAP/TAZ [94]. With the presence of LATS, ERα is targeted for ubiquitination and subsequently degraded by DCAF1-dependent proteasomes, and thus the deletion of LATS stabilizes ERα and YAP/TAZ [95].

#### Regulating other genes

Furthermore, ERα orchestrates YAP activity through its target genes. ERα forms a complex with FOXA1 and GATA3, which activates the expression of arylsulfate D (ARSD), a downstream gene of ERα. Subsequently, ARSD inhibits YAP signaling to suppress tumor proliferation and migration [96].

#### Regulating YAP nuclear translocation

Upstream Hippo components exert dual regulatory effects on ERα and YAP, such as the upstream suppressors of YAP: LATS and Ras-associated domain family 1 subtype A (RASSF1A). LATS2 co-localizes with ERα within the nucleus, and regulates ERα-driven gene transcription through direct and/or indirect interactions with ERα [97]. RASSF1A further integrates these pathways by enhancing LATS-mediated YAP inhibition to suppress ERα-dependent tumorigenesis [98].

Therefore, the interaction between YAP and ERα in breast cancer tissue cannot be understood as a simple synergy. These paradoxical observations highlight the necessity for spatially resolved analyses to delineate how Hippo-ERα crosstalk varies across tumor subclones and metastatic niches. Future investigations should prioritize identifying molecular switches that determine YAP’s dual role in ERα ^+^ breast cancer, potentially informing combinatorial therapies targeting these interconnected pathways.

### Crosstalk between Hippo-YAP and ER family across diverse tissues

While current understanding of ERα-YAP/TAZ interaction remains predominantly focused on breast cancer, other members of ER family, such as ERβ and GPCR, are also discussed in this paragraph. This section synthesizes recent findings from endometrial, myometrial, cartilaginous, hepatic, and trophoblast systems, highlighting the tissue-specific complexity of Hippo-estrogen signaling interplay.

The endometrium demonstrates dynamic regulation of YAP expression through estrogen-mediated pathways. ERα and ERβ are encoded by *ESR1* and *ESR2*, respectively, which play key roles in the regulation of endometrial and endometriosis growth [99]. Cyclic YAP expression patterns correlate with E2 fluctuations, with ER-dependent phosphorylation mechanisms modulating YAP activity [100]. Supporting this concept, Plewes *et al*. [101] proved that knockdown of *YAP1* in bovine ovarian granulosa cells (GCs) inhibited follicle stimulating hormone (FSH)-induced E2 synthesis, suggesting the importance of YAP1/TAZ in GC proliferation and E2 synthesis. Paradoxically, the reduction of YAP1 level in ovarian endometriosis enhances ESR2 expression by recruiting nucleosome remodeling and histone deacetylase (NuRD) to form the YAP1-NuRD complex and bind to the promoter of *ESR2* [99].

Mechanistic insights from uterine tissues further illuminate the Hippo-estrogen axis in fibrotic pathophysiology. Both biomechanical stress and E2 stimulation promote YAP nuclear translocation in myometrial cells, driving extracellular matrix deposition through pathways implicated in leiomyoma pathogenesis [83]. Complementary hepatic studies identify a protective regulatory loop where ginsenoside Rg1 enhances YAP expression via ERα activation, subsequently ameliorating ischaemia-reperfusion injury through oxidative stress reduction [82]. These findings collectively position Hippo-ERα crosstalk as a critical modulator of cellular stress responses across epithelial and stromal compartments.

The expanding role of membrane-associated estrogen signaling is exemplified by GPER-YAP interactions in mechanical transduction pathways [102]. Articular cartilage studies reveal a mechanoprotective axis where mechanical loading induces cytoplasmic YAP translocation, downregulating Rho GTPase-activating protein 29(ARHGAP29). ARHGAP29 is known as a regulator of the RhoA/LIMK/coflin pathway [103]. GPER inhibits mechanical stress-mediated the RhoA/LIMK/coflin pathway as well as actin polymerization by promoting the expression of YAP and ARHGAP29, as well as YAP nuclear localization, ultimately leading to inhibition of Piezo1, thereby reducing chondrocyte apoptosis [104]. In human trophoblast cells, GPER promotes human trophoblast invasion by upregulation of YAP-mediated angiopoietin-like 4 (ANGPTL4), promoting placental invasion and potentially mitigating preeclampsia risk [105]. In gastric signet-ring cell carcinoma (GSRC), the activation of GPER facilitates tumor proliferation by YAP nuclear translocation. Mechanistically, GPER inhibits LATS1-mediated YAP phosphorylation by competitively binding to ARRB2, thereby enhancing YAP activity. Meanwhile, YAP binds to the *GPER* promoter, thus forming a positive feedback loop that reinforces oncogenic signaling [106].

In hepatocellular carcinoma (HCC), Jeon *et al* [107] proved that YAP is significantly suppressed and the expression of ERα enhances YAP phosphorylation, thus promoting its nuclear translocation, which in turn suppresses the downstream signaling pathways and cancer cell growth. This research indicates that ERα expression is an indicator of more favorable prognosis in HCC and that this effect is mediated by inactivation of YAP signaling. These mechanoregulatory pathways underscore the contextual duality of YAP signaling acting as both force sensor and endocrine mediator in estrogen-responsive tissues.

## Summary and Prospect

YAP/TAZ and ERα signaling constitute two pivotal axes governing skeletal homeostasis through distinct yet interconnected mechanisms. The mechanosensitive YAP/TAZ module operates via Hippo-dependent (LATS kinase-mediated phosphorylation) and Hippo-independent (cytoskeletal tension/nuclear mechanosensing) pathways to translate biomechanical cues into transcriptional programs directing osteoblast-osteoclast equilibrium. Complementarily, ERα integrates endocrine signals through genomic (nuclear receptor-DNA binding) and non-genomic (membrane-initiated kinase cascades) modalities, while exhibiting mechanoresponsive properties through ligand-independent phosphorylation cascades. Crucially, emerging evidence positions ERα as a molecular nexus where mechanical and estrogenic signals converge to regulate RUNX2-mediated osteogenesis and RANKL-dependent osteoclastogenesis.

Despite their individual importance, critical knowledge gaps persist regarding ERα-YAP/TAZ interplay in bone biology. The stage-specific cooperation/competition between these pathways during osteoblast differentiation remains undefined. Preliminary data suggest that YAP/TAZ may inhibit early osteoprogenitor commitment while enhancing mature osteoblast activity, a pattern potentially modulated by ERα status. Paradoxically, the best-characterized ERα-YAP/TAZ interactions derive from breast cancer studies, revealing context-dependent synergism (enhancer co-occupancy, epigenetic regulation) and antagonism (competitive TEAD binding, proteasomal degradation). While these models provide conceptual frameworks, direct extrapolation to bone is precluded by tissue-specific factors. YAP plays a dual role in inducing apoptosis and inhibiting tumor growth. ERα and YAP proteins can directly affect each other’s expression by acting on gene elements, and can also interact with upstream molecules in the pathway such as LATS, MST, RASSF1A, or indirectly by affecting intermediate genes such as *ARSD*. However, a few studies have suggested that YAP and ERα are antagonistic to each other in breast cancer. Therefore, the interaction between YAP and ERα in breast cancer tissue cannot be understood as a simple synergy, considering the complexity of the components of Hippo-YAP pathway, the relationship between YAP and ERα in breast cancer tissue should be carefully considered because of the multiple and even contradictory effects of different components in breast cancer cells. Addressing these challenges requires a multidisciplinary approach, and further research directions should focus on advanced mechanobiology models like 3D osteogenic scaffolds with tunable stiffness and estrogen gradients for dissecting pathway crosstalk under physiomimetic conditions. Spatiotemporal mapping studies like single-cell RNA-seq/ATAC-seq profiling of ERα-YAP/TAZ targets across osteoblast differentiation trajectories under combined mechanical/estrogenic stimulation are also necessary. In summary, the ERα-YAP/TAZ axis represents a prototypical mechanohormonal signaling network where extracellular forces and endocrine cues coalesce to maintain skeletal integrity. Deciphering their crosstalk mechanisms will not only advance fundamental understanding of bone biology but also unveil “two-for-one” therapeutic strategies targeting both mechanical insufficiency and hormonal imbalance in osteoporotic bone loss. As mechanical loading regimens and estrogen-based therapies currently operate in clinical silos, elucidating their molecular convergences could catalyze transformative combinatorial approaches for skeletal rehabilitation.
